# The Adhesive System and Anisotropic Shear Force of Guizhou Gastromyzontidae

**DOI:** 10.1038/srep37221

**Published:** 2016-11-16

**Authors:** Jun Zou, Jinrong Wang, Chen Ji

**Affiliations:** 1 State Key Laboratory of Fluid Power and Mechatronic Systems, Zhejiang University, Hangzhou 310027, China

## Abstract

The Guizhou gastromyzontidae (*Beaufortia kweichowensis*) can adhere to slippery and fouled surfaces in torrential streams. A unique adhesive system utilized by the fish was observed by microscope and CLSM as an attachment disc sealed by a round belt of micro bubbles. The system is effective in wet or underwater environments and can resist a normal pulling force up to 1000 times the fish’s weight. Moreover, a mechanism for passive anisotropic shear force was observed. The shear forces of the fish under different conditions were measured, showing that passive shear force plays an important role in wet environments. The adhesive system of the fish was compared with other biological adhesion principles, from which we obtained potential values for the system that refer to the unique micro sealing and enhanced adhesion in a wet environment.

The Guizhou gastromyzontidae (*Beaufortia kweichowensis*) is a species distributed throughout southwest China. This fish has developed an adhesive disc, enabling it to adhere to wet and slippery rocks, holding it in position in torrential streams with high, variable forces. Other fishes with adhesive capabilities, such as the clingfish (*Gobiesocidae*), were reported to adhere to surfaces with a wide range of roughness^1^. Previous work attributed the effective adhesion of clingfish and remora fish to the hierarchically structured microvilli around the edges of the adhesive disc^1^,^2^,^3^,^4^. The hierarchical structure helped the attachment disc to interdigitate with objects, especially on rough surfaces. In addition to the clingfish, adhesion based on microstructures has been widely reported in the literature^5^,^6^. The most famous example might be the van der Waals adhesion of geckos^7^,^8^,^9^, while many other species, such as snail^10^, tree frog^11^,^12^, abalone^13^ and insects^14^,^15^, have also been reported to adhere with various mechanisms in wet or underwater environments. The diversity and efficiency of biological adhesion make it a very attractive topic in terms of both science and practice.

In the present work, we experimentally investigated the adhesion of *B. kweichowensis* (sometimes noted as “the fish” in the following sections), with an adhesive disc similar with the majority of gastromyzontine loaches. The structure and adhesive mechanism of gastromyzontine loaches have been reported since the 1930s^16^,^17^,^18^. The fishes depend on a suction disc to attach to rocks, and the small keratinous structures covering the surface of the fins and body, unculi^18^, are important to increase friction. In the literature, increasing the friction factor was considered to be the primary function of the unculi^18^,^19^, but there was not any functional evidence^16^. Based on experimental results, we describe a unique adhesive system of *B. kweichowensis*. Stable micro bubbles on the ventral side of the fish were observed by microscope and CLSM (confocal laser scanning microscopy). Based on force measurements, the primary function of the unculi seems to be to entrap micro bubbles to seal the attachment disc. Moreover, a unique phenomenon of anisotropy adhesion was observed in both experiments and the daily life of the fish, which was studied by shear force measurement and MicroCT. The experimental results show a unique adhesive system with potential values, and may fulfil the call for functional work on the role of unculi^16^.

## Results

### Unculi and micro bubbles

**Figure 1:**
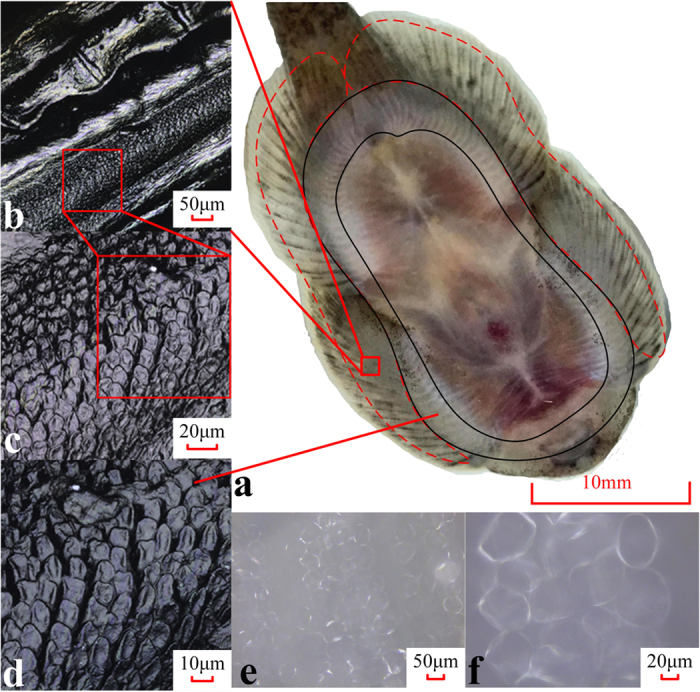
Ventral structure of the fish. (**a**) The ventral view of the fish. Unculi distribute continuously throughout the area between the black solid lines and not continuously in the area noted by the red dashed lines. (**b–d**) Microscopic images of the micro structured region, captured by CLSM (laser mode). (**e,f** ) Micro bubbles between the structured skin and the cover glass, with an average diameter of approximately 50 microns, captured by CLSM (optic mode). All images were taken after the specimens were euthanized, without any other treatment.

Figure 1(a) presents the ventral view of *B. kweichowensis*. Using CLSM, we found unculi under the fins and near the maxilla, which has been reported in literature^18^. As shown in Fig. 1(d), the unculi are columns with an average diameter of approximately 9 μm with bottoms that are slightly concave. In the area surrounded by a dashed red line (Fig. 1(a)), the unculi are observed in the gaps between the fin rays, as shown in Fig. 1(b,c). At the root of the fins (noted by a solid black line in Fig. 1(a)), similar micro columns are distributed continuously, as shown in Fig. 1(d).

Particularly when the ventral side of the fish is wet, a different pattern appears in the area containing unculi due to the formation of micro bubbles. Microscopic pictures were taken when the fishes were attached to a cover glass with a water film, as shown in Fig. 1(e,f), where the bubbles are clear, and the unculi appear to not contact the substrate. The bubbles have an average size of 50 μm, about five times larger than the micro columns.

### Anisotropic shear force of the fish

**Figure 2:**
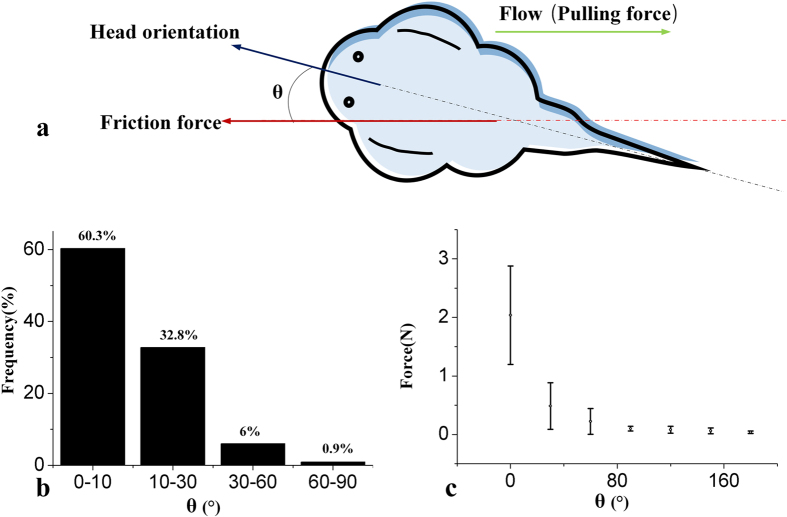
Life habit and anisotropic shear force of the fish. (**a**) Definition of *θ*; (**b**) distribution of *θ* after the fish is washed by a random horizontal jet; (**c**) the relationship between the friction force and *θ.*

In a rushing stream, the friction in the opposite direction of the dragging force helps the fish to fix itself. In these experiments, we found the fish utilizing anisotropic shear force. As shown in Fig. 2(a), *θ* is defined as the angle between the direction of the fish head and the shear force acted on it. First, we created a water jet from a random direction towards 4 living fishes 29 times for each fish. Figure 2(b) shows that in most cases, *θ* is less than 10 degrees, indicating that the fishes tend to turn their head against the coming water flow. Further results of the maximum shear force in different directions are shown in Fig. 2(c). With *θ* changing from 0° to 180°, the maximum shear force decreases dramatically. This anisotropic shear force explains the directional preference of the fish, which may also help the fish to switch between sound adhesion and rapid detachment.

**Figure 3:**
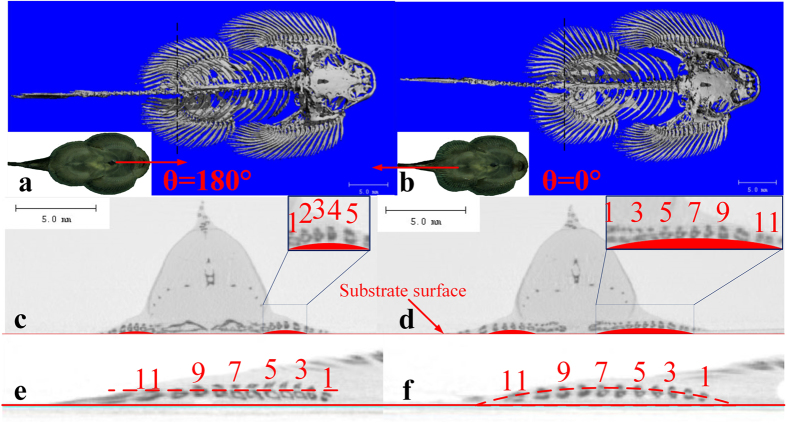
Micro CT comparison of different conditions of the fins. (**a**) Fish pulled with *θ* = 180°; (**b**) fish pulled with *θ* = 0°; (**c,d**) the cross profile of the fish at the black dashed lines, referring to (**a**) and (**b**), respectively; (**e,f** ) the height of the fin ray roots, referring to (**a**) and (**b**), respectively. Pictures in (**a–f**) are captured by MicroCT and processed in Mimics Medical 17.0.

To reveal the cause of the anisotropic suction pressure and friction, a microCT scan^20^ was carried out. Two different statuses of the fins could be obtained from the CT results, as shown in Fig. 3. When the fish is pulled from the head (*θ* = 180°), the pectoral fins and ventral fins are adducted, leading to a smaller area of fins from the top view, as shown in Fig. 3(a). On the contrary, the pulling with *θ* = 0° makes the fins expand (Fig. 3(b)). On the side slice view, the case of *θ* = 0° lifted more fins to detach from the substrate. Comparing Fig. 3(c,d), only 4 fin rays were not contacting the substrate in the status of adduction, while in the expansion status we found 8 fin rays arisen from the substrate. Moreover, in Fig. 3(e) the roots of the 11 rays had a similar height, while Fig. 3(f) shows that several roots rose up in the case of *θ* = 0°. In this way, the horizontal force causes a passive volume change of the attachment disc, which depends on the dragging direction.

### Adhesive force and shear force

**Figure 4:**
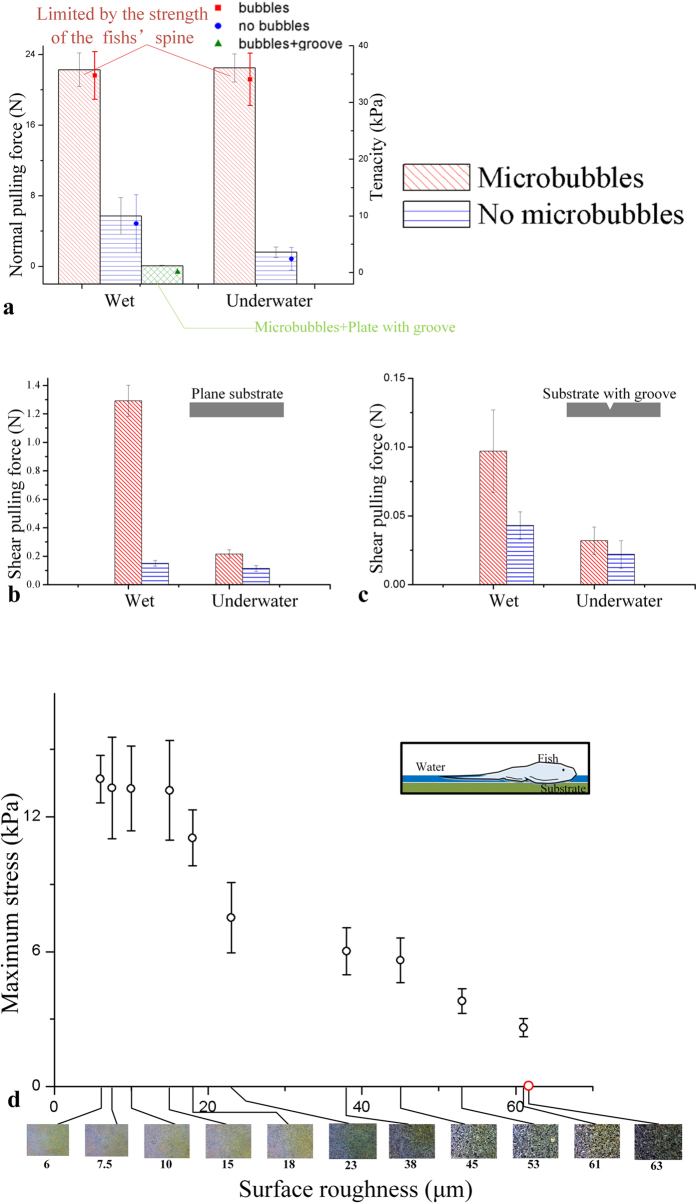
Normal adhesion forces/tenacities and shear pulling forces on wet and underwater substrates. (**a**) The squares show the normal adhesive force, and the points represent the adhesive tenacity; (**b**) the shear force on a smooth plane plate; (**c**) the shear force on a plate with a groove. (**d**) Tenacities on different rough surfaces. The pictures below the graph are the substrates with different roughness, captured by a digital microscope (Qiyuan 40–800 Zoom). The fish could adhere to all the substrates except the last one.

The adhesive ability of the fish was tested on smooth, transparent acrylic plates, as shown in Fig. 4. The weights of the fishes ranged from 1.81 g to 2.89 g, with attachment disc areas between 654 to 895 mm^2^. The adhesive forces were measured under two conditions: wet and underwater. In the underwater case, the fish was first put on the substrate in air and then moved slightly into the water tank to maintain the film thickness between the fish and the substrate.

The results show that micro bubbles play an important role in adhesion. As shown in Fig. 4(a), the normal adhesive force is negligible on the plate with a groove, indicating the suction effect is of the most importance. In both the wet and underwater cases, micro bubbles form under the fins led to an impressive adhesive ability. The maximum normal force recorded was over 22 N, with tenacity larger than 30 kPa, which broke the spine of the fish before detachment from the substrate. For comparison, we put the fish in ethanol to dissolve the micro bubbles. The fish was submerged into solutions of 25%, 50%, 75%, 95% ethanol and pure ethanol in sequence (10 min for each solution). During the treatment, the fish was dried. The unculi could recover after the fish was re-submerged in water for 10 minutes, but the micro bubbles disappeared. In tests of fishes without micro bubbles, the maximum normal force decreased dramatically.

In tests of shear force, an extra load of 30 g was exerted on the fish, and in all tests, we pulled the fish in the direction of *θ* = 0°. Figure 4(b) presents the results on the groove-less substrate. The fish with micro bubbles could resist a horizontal pulling force up to 1.3 N in the wet case, while the value reduced to 0.15 N in the underwater tests. In contrast, the shear force without the micro bubbles maintained a low value in both the wet and underwater cases. In addition, a substrate with a groove was employed to obtain shear despite the suction effect. As shown in Fig. 4(c), the variation of the shear forces is similar to Fig. 4(b). However, the shear force in the wet case is only 0.34 N. Compared with Fig. 4(b), the shear force difference in Fig. 4(c) is much smaller.

By using wet sandpapers as the substrates, we tested the ability of *B. kweichowensis* to attach to rough surfaces. As shown in Fig. 4(d), the adhesive tenacity decreased on rougher substrates. The fish failed to adhere to sandpapers with a surface roughness larger than 63 microns.

## Discussion

**Figure 5:**
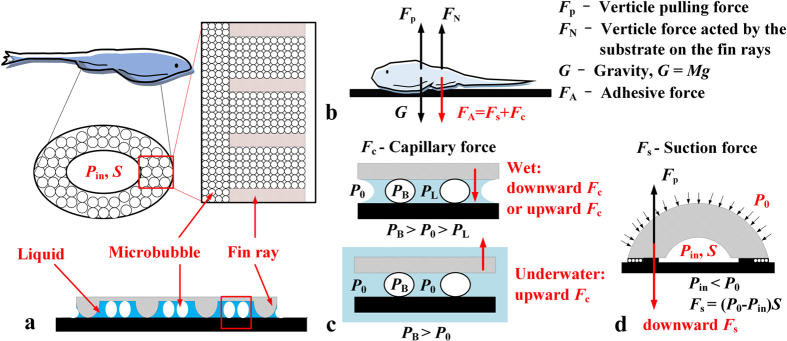
The adhesive system. (**a**) The sketch of the fin region; (**b**) the normal forces acted on the fish when the fish is pulled upward; (**c**) the suction force *F*_s_.

*B. kweichowensis* lives in the torrential streams with rapid and variable flows. The fish usually rests still on horizontal, oblique or vertical surfaces of the rocks. The fish is also accustomed to climb its way upstream, which can also be observed in lab environment. Therefore, the ability of firmly attaching to substrate and resisting high shear force is strongly relevant for the fish. Based on our observations, a sketch of the adhesive system of *B. kweichowensis* is shown in Fig. 5(a). The major adhesive force comes from the low pressure *P*_in_ in the attachment disc with an area of *S*, which is surrounded by a belt of micro-structured skin. The region with unculi (~10 microns) seals the attachment disc, and the fin rays between the unculi regions produce friction in the tangential direction. Specifically, micro bubbles (~50 microns) form when the structured region is wet, which proved to be essential for the fish to resist both the normal pulling force and the tangential drag force. The micro bubbles may form by short exposure to the air, from dissolved gas in the water, or from gas released by the fish. The bubbles were observed beneath all of the tested fishes, and we found that they seldom disappeared naturally, even after the fishes were submerged for several days.

The attachment disc of *B. kweichowensis* almost covers the entire ventral side of the fish, formed by the pectoral fins, the pelvic fins and the oral tissue. For the 12 fish we tested, the body length and weight ranged from 5.1 cm to 7.3 cm and from 1.81 g to 2.89 g, respectively. The area of the attachment disc ranged from 654 mm^2^ to 895 mm^2^. In comparison to other fish species with attachment discs, such as remoras^4^,^21^, clingfishes^1^,^22^ and gobbies^23^,^24^, the attachment disc of *B. kweichowensis* is very large in relation to the body. In addition, as shown in Fig. 4(a), the maximum tenacity exceeded 30 kPa on a wet smooth substrate, noting that the measurement was limited by the strength of the spine. In the species with similar attachment discs, remora had the largest tenacity of up to 100 kPa^21^. The tenacity of *B. kweichowensis* is similar to the clingfish (approximately 30 kPa on smooth surface^1^), but much higher than the climbing goby *Sicyopterus stimpsoni* and the non-climbing goby *Stenogobius hawiiensis*^23^. A large disc with a high adhesive tenacity can produce an impressive normal adhesive force, up to 1000 times larger than the fish’s weight. However, a larger disc may reduce the ability of the fish to attach to rough surfaces. As shown in Fig. 4(d), the tenacity goes down with larger surface roughness, indicating that *B. kweichowensis* cannot adapt to rugged substrates as well as the clingfish^1^,^22^.

In some published works, the unculi were supposed to be a key factor to prevent slipping^17^,^18^,^19^. However as reviewed by Conway *et al*.^16^, there was no functional evidence for the role that unculi actually perform. From Fig. 1, we can relate the unculi to the micro bubbles beneath the fish. As shown in Fig. 4, the micro bubbles play an important role in the adhesion system, but the function of the micro bubbles beneath the fish has never been reported in literature. Therefore, the functional mechanism of both the unculi and the micro bubbles is of interest. Based on the adhesion effects reported previously, we can hypothesize four possible mechanisms for the unculi and the micro bubbles: (A) The contact splitting effect^25^ of the unculi; (B) The wet adhesion effect, induced by the capillary bridges^26^ between the micro bubbles; (C) The micro bubbles help to seal the suction disc; and (D) The unculi increase the friction factor of the fish to prevent slipping.

In order to reveal the function of the unculi and the bubbles, an analysis of the normal forces is necessary. Figure 5(b) shows the normal forces acted on a fish being pulled upward by an external force *F*_p_ (*F*_p_ is the pulling force measured in Fig. 4(a)). The adhesive force *F*_A_ represents the normal force produced by the adhesive system, which could be further divided as the capillary force *F*_c_ and the suction force *F*_s_. The substrate also acts a normal force of *F*_N_ to balance the forces. The basic principle of suction force is shown in Fig. 5(c). A negative gauge pressure *P*_in_ is induced by pulling the fish upward, producing a downward suction force *F*_s_ = (*P*_0_ − *P*_in_)*S*. Note that the suction disc is filled with liquid in wet and underwater cases, thus a slight expansion of the disc may lead to a very low *P*_in_.

Based on the force analysis, we can firstly exclude hypothesis (A) because the microscopic pictures negate a situation of direct contact between the unculi and the substrate, as shown in Fig. 1(e,f). Moreover, the size of the unculi (~10 μm) is too large to produce an adhesive force high enough. On the other hand, the capillary force *F*_c_ in hypothesis (B) does exist in wet cases, or in underwater cases with micro bubbles. The effect of *F*_c_ can be observed in Fig. 4(c), where *F*_c_ caused a decrease of 0.19 N of the shear force in the underwater case. However as shown in Fig. 4(a), when a groove on the substrate was induced to remove *F*_s_, the maximum *F*_p_ decreased dramatically even though the micro bubbles still exist. Therefore *F*_c_ 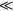 *F*_s_, indicating that the wet adhesion effect is not the primary mechanism, and the hypothesis (B) cannot explain the function of the micro bubbles.

By comparing the adhesive force with and without micro bubbles on wet substrates, we can deduce that hypothesis (C) describes the primary function of the micro bubbles. As shown in Fig. 5, the fin rays of the fish produce the friction, while the unculi under the fins are separated from the substrate by a liquid film. Without the micro bubbles, the suction performance depends on the Stefan adhesion effect of the liquid film, affected by the viscosity. As shown in Fig. 4(a), this weakly sealed attachment disc could resist a normal pulling force up to 8 N. Upon inducing the micro bubbles, the maximum suction force rises to over 20 N, even able to break the spine of the fish. We also found that the bubbles allowed the fish to adhere much more easily. The adhesion of the fish with micro bubbles does not need pressure, as slightly putting it on the substrate led to sound adherence, while extra pressing was necessary for the adhesion of the fish without bubbles.

The sealing effect of the micro bubbles could be explained by the increase of fluidic resistance in the micro gap between the fin and the substrate. The pressure drop of the two-phase gas/liquid flow is a complex problem, affected by many factors including the flow rate, the passage geometry and the two-phase flow pattern^27^,^28^. In the experimental study of homogeneous gas/liquid flows in square micro channels, Cubaud^29^ reported that inducing the gas phase could lead to a larger pressure drop. The condition for an increase in fluidic resistance was a low velocity (<40 mm/s), a gas fraction of less than 80%, and a flow pattern with partial wetting effect. In the present case, the micro bubbles and liquid bridges formed a partial wetting pattern, while the flow velocity in the gap was quite low. Although the flow in the gap was not homogeneous, it is a reasonable deduction that the bubbles helped the attachment disc to hold up a larger pressure difference, leading to a larger adhesive tenacity in wet or underwater environments.

Shear force is also an interesting topic, especially in wet or underwater adhesion scenarios^30^,^31^. When the fish is moving because of a shear pulling force, the primary force to resist the pulling force is the sliding friction *μF*_N_, where *μ* is the coefficient of kinetic friction. In wet or underwater cases, we can expect a shear force way smaller than the normal suction force, because *μ* decreases with the liquid film lubrication. As shown in Fig. 2, we observed a unique anisotropic shear force in the fish. The expansion of the fins provided a passive decrease of *P*_in_, leading to a higher *F*_N_ and an additional friction. In Fig. 4(b), the expansion of the attachment disc was obstructed by the extra load. Although the load was more than 10 times the weight of the fish, the shear force was still less than that in Fig. 2(c) without any extra load, indicating the importance of anisotropic shear force. This mechanism coincided with the habit of the fish. The passive effect helped the fish to adhere easily when it heads upstream to feed, while it did not work when the fish was swimming forward.

In previous works, the unculi were supposed to be a key factor to prevent slipping^17^,^18^,^19^, and the hypothesis (D) above was applied to explain the mechanism. However, Fig. 4(b,c) showed that the shear force always decreased after the micro bubbles were removed. We noted that treatment with ethanol had a drying effect on the unculi, but in cases “without micro bubbles” in Fig. 4, the dried fish was re-submerged in water for 10 minutes and the unculi recovered. According to this hypothesis (D), the fish should resist a similar shear force with or without the micro bubbles. Therefore, increasing the friction factor is only part of the function of the unculi. As shown in Fig. 4(b), a dramatic increase in friction was observed in the wet case. The friction is produced by a combination of the suction force and the capillary force, both of which are related to the micro bubbles. We deduced that the primary function of the unculi of *B. kweichowensis* is to prevent the bubbles from detaching and to seal the attachment disc. With the mechanism of the anisotropic shear force mentioned before, the sealed disc can resist a large shear force passively.

As noted previously, when the sealing effect of the micro bubbles is employed, an extra 30 g load weakens the shear force. However, in the underwater case, the fish with micro bubbles could only hold a shear force of 0.2 N. To explain the decrease of the friction underwater, we suppose that the passive expansion of the attachment disc depended on the pulling force acting on the fish. In the underwater case, the friction produced by gravity and capillary force was much smaller than in the wet case. Therefore, a small shear force may cause a horizontal slip, preventing the pulling force from rising further to expand the fins. In a natural habitat, a living fish can avoid the slip by squeezing the attachment disc with the fins.

In conclusion, our results demonstrate the unique adhesive system of the gastromyzontidae, and provide experimental evidence for the function of the unculi. The unculi and bubbles help to seal the attachment disc, producing impressive adhesive force with the aid of passive anisotropic shear force. The adhesive system is very effective in wet and underwater environments and helps the fish to detach rapidly from the rocks. This mechanism may be a possible biomimetic solution to the problem of reversibly adhering to submerged surfaces with anisotropic shear force. However, further questions need to be investigated in further works, including the mechanism to form and fix the micro bubbles with a certain size and the pressure drop model with bubbles fixed in the gap.

## Methods

### Specimens

The experiments herein were approved by the animal welfare committee of Zhejiang University, and all the experimental trails were carried out in accordance with the relevant guidelines and regulations.

22 specimens of *B. kweichowensis* were used in the experiments that were caught in the rocky torrential streams of Yangshuo, Guangxi Province. The fishes lived together in an aquarium with an oxygen pump, a heater and a filter, where the temperature was kept at 26 °C. The aquaria were covered with loose sand substrate with a depth of approximately 5 cm, together with some stones on which the fish could rest. Usually, the fish adhered to the stones or the aquarium walls, and only occasionally they moved to a different location. Before the experiments, the fish were fed three times a week with fish food. The fish were euthanized^1^ via submersion in a concentration of 0.5 g/L MS 222 for 15 minutes, then weighed and photographed for later measurement of length and attachment disc area. The length of the fish ranged from 5.1 to 7.3 cm. The weights of the fish ranged from 1.81 to 2.89 g, with attachment disc areas ranging from 654 to 895 mm^2^. The 4 fishes used for jet tests were from 5.2 to 7.2 cm long, 1.86 to 2.83 g weight. The 10 fishes used for adhesive force tests on smooth surface were from 5.1 to 7.3 cm long, 1.84 to 2.89 g weight, with disc areas from 654 to 895 mm^2^. The 3 fishes used for shear force tests were all 5.2 cm long, 1.86 to 1.90 g weight, with disc areas from 668 to 674 mm^2^. The 5 fishes used for adhesive force tests on rough surface were from 5.1 to 7.0 cm long, 1.81 to 2.76 g weight, with disc areas from 654 to 873 mm^2^. The surface area of the attachment discs was measured from digital images in IMAGEJ (NIH, available at http://rsbweb.nih.gov/ij/).

### Force measurements

We performed force measurements with the fresh euthanized fish. For force measurements, we used a digital force gauge (HBO HF-50) with a screw platform system at a constant pulling speed of 13.5 mm/s. When force measurements were applied in the water, a fixed pulley 24 mm in diameter was needed to adjust the cord direction. The substrates employed in the smooth surface experiment were two types of transparent acrylic plates: (1) a smooth 10 cm × 10 cm plane transparent acrylic plate; and (2) a 10 cm × 10 cm smooth transparent acrylic plate with a 5 mm deep and 5 mm wide groove in the middle. In the test of adhesive ability on rough substrates, the substrates employed were 11 different sandpapers (10 cm × 10 cm) inlaid in epoxy resin with roughness of 6, 7.5, 10, 15, 18, 23, 38, 45, 53, 61 and 63 microns.

**Figure 6:**
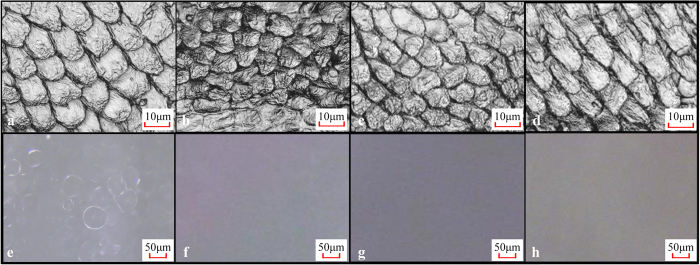
Comparison of fresh, dried and recovered fishes. (**a–d**) Unculi, taken by CLSM (laser mode); (**e–h**) micro bubbles, taken by CLSM (optic mode); (**a**) and (**e**) fresh euthanized fish; (**b**) and (**f**) fish dried by ethanol solutions; (**c**) and (**g**) recovered fish (exposed to air); (**d**) and (**h**) recovered fish (not exposed to air).

**Figure 7:**
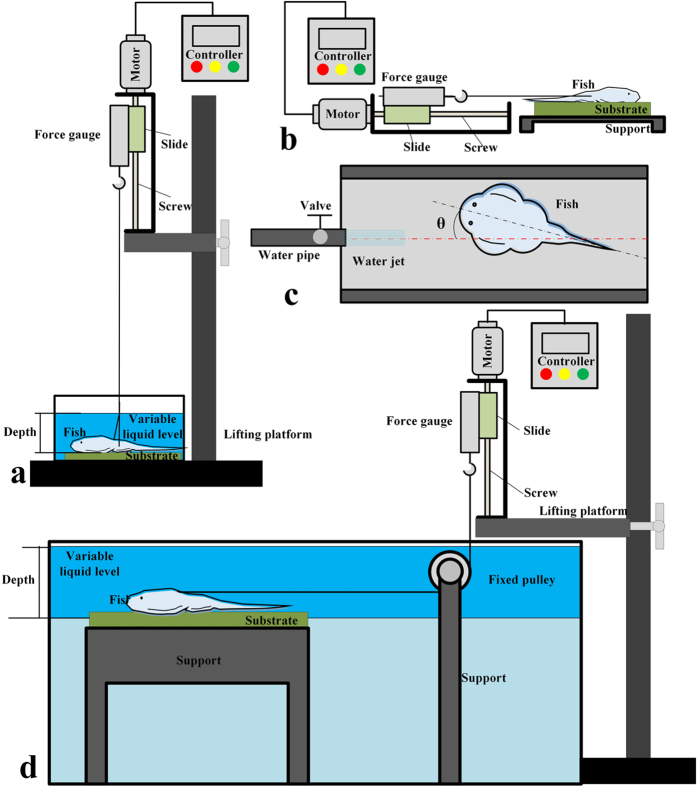
Illustration of the experimental set up for force measurement. (**a**) Normal adhesive force test; (**b**) shear force test; (**c**) life habit test; (**d**) shear force test underwater.

Fresh, euthanized fish (Fig. 6(a,e)) were put on the substrate, which was covered by a 1 mm thick water film in the wet cases. In underwater cases, the fish were put on a wet substrate first, then moved into a water tank with a depth of 6 cm. In some experiments, the fish were put into 25% ethanol, 50% ethanol, 75% ethanol, 95% ethanol and 100% ethanol in sequence for 10 min each to dehydrate the specimens (Fig. 6(b,f)), then were processed in inverted sequence, at last put into distilled water for 20 min to dissolve the micro bubbles. As shown in Fig. 6(d) and (h), the unculi of the “recovered” fish were similar to the fresh fish, but the micro bubbles almost disappeared completely. In Fig. 6(c,g), the fish was exposed to air for 30 seconds before put into distilled water, showing that the unculi and the micro bubbles were rarely affected by the exposing process. After the drying and recovery process, the fish was put on a substrate and depressed gently to evacuate some water under the disc (preliminary experiments showed that further pressing did not lead to larger adhesive forces), then the fish was hooked up. We pulled the fish in a normal direction to test the adhesive force (Fig. 7(a)) or pulled it in different shear directions to measure the shear force (Fig. 7(b,d)). 5 trials of each test recorded adhesive force or shear force. In the measurement of the relationship between shear force and pulling angle, 3 trails were performed for each angle.

### Water-jetting tests

To test the fish’s natural reaction to the water flow, a living fish was randomly put on a smooth substrate in the aquarium, then a water jet of 100 ml/s was produced by a nozzle towards the fish (Fig. 7(c)). After 1 minute, we recorded the angle between the direction of the jet and the fish. The fish was then re-set on the substrate and recovered for 1 min before being washed again. In this experiment, four fish were tested, repeating 29 times for each fish.

### Microscopic imaging

A digital microscope (Qiyuan 40–800 Zoom) and a tabletop CLSM (KEYENCE VX-150X) were employed to observe the epithelial microstructure of the fish’s ventral side. Epoxy resin support was used to make sure the upper surface under the microscope was flat. The micro bubbles formed under the fish were observed using the optic mode of the CLSM when the fish was attached to a cover glass with a water film between them. Microstructures were observed using the laser mode of the CLSM when the fish was upside-down towards the microscope lens. We observed the fresh euthanized specimens, the dehydrated specimens and the water-recovered specimens using the laser mode of the CLSM and the digital microscope to estimate the shrinking effects. The substrate surfaces were observed with the digital microscope.

### MicroCT scan

MicroCT (SCANCO MEDICAL μCT 100) was used to scan the euthanized fish to observe the inside structure when the fish was pulled from the direction of the head or tail. During the scanatron, the euthanized fish adhered to horizontal, smooth cardboard fixed to a bar of foam PET. Fish were scanned with a resolution of 35 μm at a source voltage of 100 kV at a current of 200 μA. The data were analysed in Mimics Medical 17.0.

### Data analysis

In each trial, the adhesive tenacity *δ* was calculated as *δ* = *F*/*A*, where *F* is the normal pulling force, and *A* is the area of the specimen’s attachment disc.

For statistical analysis of the data, we performed five, two-way ANOVA (analysis of variance) and one, one-way ANOVA with Microsoft Excel 2013. We evaluated the effect of single parameters on our results by analysing the ANOVA table, and the level of significance was set to α < 0.01. We tested six parameters: (1) the adhesive force on the smooth surface as a dependent variable with the effect of micro bubbles and the conditions of the specimens (wet/underwater); (2) the shear force on the smooth surface as dependent variable with the effect of micro bubbles and the conditions of the specimens; (3) the shear force on the surface with a groove as dependent variable with the effect of micro bubbles and the conditions of the specimens; (4) the stable angle of the living fish’s direction variable with the individual fish and the flow angle; (5) the shear force of the fish variable with the individual fish and the staying angle; and (6) the adhesive force on the rough surface variable with the surface roughness of the substrates. Based on the ANOVAs, we performed a P-value test for statistical significance (p < 0.01), as shown in Table 1.

## Additional Information

**How to cite this article**: Zou, J. *et al*. The Adhesive System and Anisotropic Shear Force of Guizhou Gastromyzontidae. *Sci. Rep.* **6**, 37221; doi: 10.1038/srep37221 (2016).

**Publisher’s note:** Springer Nature remains neutral with regard to jurisdictional claims in published maps and institutional affiliations.

## Figures and Tables

**Table 1:** ANOVA results of the data.

ANOVA table for variance analysis			
Independent variable	DF	F value	p value
Response variable: the adhesive force on the smooth surface			
Condition	1	45.3575	**4.79E-06**
Bubble	1	4187.917	**8.66E-21**
Interaction	1	55.44147998	**1.3893E-06**
error	16		
Response variable: the shear force on the smooth surface			
Condition	1	10447.36364	**9.38E-14**
Bubble	1	13196.45455	**3.69E-14**
Interaction	1	9251	**1.52E-13**
error	8		
Response variable: the shear force on the surface with a groove			
Condition	1	188.4615385	**7.6438E-07**
Bubble	1	199.3846154	**6.1507E-07**
Interaction	1	88.61538462	**1.3303E-05**
error	8		
Response variable: the stable angle of the living fish’s direction			
Fish individual	3	0.209302	**0.887443**
Flow angle	3	203.7907	**1.37E-08**
error	9		
Response variable: the shear force of the fish in different direction			
Fish individual	3	3.923587	**0.025648**
Staying direction	6	42.42719	**5.00E-12**
error	18		
Response variable: the adhesive force on the rough surface			
Surface roughness	9	47.27707705	**1.59E-18**
error	40		
